# Mosquito bite prevention through self-assembled cellulose nanocrystals

**DOI:** 10.1093/pnasnexus/pgad069

**Published:** 2023-04-11

**Authors:** Daniel Voignac, Evyatar Sar-Shalom, Yossi Paltiel, Oded Shoseyov, Jonathan Bohbot

**Affiliations:** The Robert H. Smith Institute of Plant Sciences and Genetics in Agriculture, Food and Environment, The Hebrew University of Jerusalem, Rehovot 7610001, Israel; Department of Applied Physics and Center for Nanoscience and Nanotechnology, The Hebrew University, Jerusalem 9190401, Israel; The Robert H. Smith Institute of Plant Sciences and Genetics in Agriculture, Food and Environment, The Hebrew University of Jerusalem, Rehovot 7610001, Israel; Department of Applied Physics and Center for Nanoscience and Nanotechnology, The Hebrew University, Jerusalem 9190401, Israel; The Robert H. Smith Institute of Plant Sciences and Genetics in Agriculture, Food and Environment, The Hebrew University of Jerusalem, Rehovot 7610001, Israel; The Robert H. Smith Institute of Plant Sciences and Genetics in Agriculture, Food and Environment, The Hebrew University of Jerusalem, Rehovot 7610001, Israel

**Keywords:** mosquitoes, bite prevention, self-assembly, nanocellulose

## Abstract

Mosquitoes are the deadliest of all combined insects and animals affecting millions and killing hundreds or thousands of people each year. Existing protection methods however are limited and include volatile compounds that actively repel mosquitoes such as N,N-Diethyl-meta-toluamide (DEET) or different essential oils such as geraniol and citronella. Most are odorous compounds and require organic solvents for dispersion. This work investigates the barrier properties of cellulose nanocrystals (CNCs). CNCs are known to self-assemble in strong, transparent, chemical barrier films. They are fully bio-based, and their surface chemistry is ideal for aqueous dispersion of many compounds. This work saw a significant 80% decrease in feeding on human skin when a thin CNC coat was applied. The effect was further confirmed by artificial feeding on *Aedes aegypti* wherein CNC appears to act as a chemical camouflage to the many cues sought by the insects. The combined effect of CNC with indole reduced egg laying post exposure to mammalian blood close to null with 99.4% less eggs as compared to control. The chemical barrier effect was assessed through a simple headspace experiment showing that the same CNC coat blocked the passage of ammonium hydroxide vapor, a commonly used mosquito attractant, when applied on a filter paper membrane.

**Significance**

This paper presents a novel, scalable, and safe mosquito bite prevention method. Cellulose nanocrystals (CNCs) can be used as a chemical camouflage on skin. It prevents the emission of typical cues mosquitoes seek for landing and subsequent bloodfeeding. The unique nano-structure and chemistry of CNC also make it a platform for the safe and stable dispersion of existing active mosquito repellents for their sustained release.

## Introduction

Mosquitoes are the deadliest animals on earth. They are vectors of many parasitic and epidemic diseases including malaria, zika, chikungunya, yellow fever, and many more. In its latest report, the World Health Organization (WHO) estimates 241 million cases of malaria in 2020 and out of these approximately 627,000 deaths (1). The United Nations placed eradicating malaria in its top goals to be reached by 2030 (sustainability development goals (SDG) 3.3) (2). Paving the way to achieve this goal, various methods are being explored ranging from preventive vaccine (3), treatments of symptoms (4) to solutions that target bite prevention in the first place or mosquito population eradication (5). One key solution is the development of efficient personal protective equipment (PPE) including repellents to be applied on exposed skin areas. These may be natural, odorant compounds such as citronella or geranium-derived oils, or synthetic non-odorant like N,N-Diethyl-meta-toluamide (DEET) (6). These are generally active compounds that act on olfactory receptors making the emitting skin area an undesirable target to land and probe to feed (7, 8). However, these materials have a limited effective distance and time range.

This work proposes to investigate nano-biomaterials for the development of new, improved solutions for PPE against mosquitoes. A solution that does not chemically interact with the body odors but rather forms a barrier to block them and reduce the biting.

**Fig. 1.**
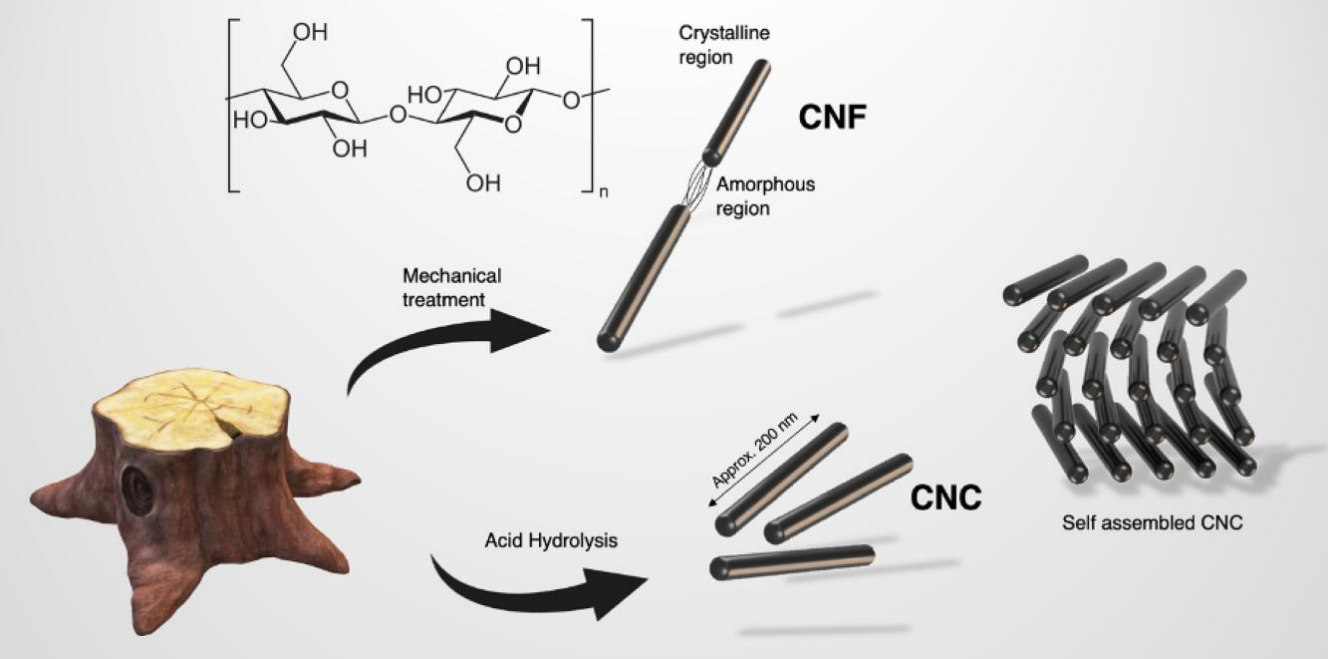
Hierarchy of cellulose from bulk wood to nanostructures.

Neves Borgheti-Cardoso et al. review the landscape of nanomaterials in the fight against malaria, suggesting different approaches in taking advantage of unique novel materials for targeted drug delivery and active fighting against the disease (9). In 2019, Castilho et al. proposed a non-chemical barrier to prevent mosquito bites using graphene. Their initial assumption was creating a physical shielding layer that would prevent the mosquito’s proboscis penetration, thanks to graphene’s outstanding strength. They report a considerable effect with no biting occurring with graphene applied on tested skin. With no biting as well as no visits from feeding mosquitoes, they were only able to conclude on a chemical barrier effect. The graphene acted as a barrier for the usual cues that trigger landing and probing (sweat, CO${}_{2}$, hormones, humidity, etc.). In the same work, they performed mechanical probing in vitro to mimic the maximum force a mosquito proboscis could apply to assess their initial claim. Putting this hypothesis to test in vivo, on a human hand, they faced difficulties pinpointing the exact mechanism. While this work is fascinating in its bioconvergence approach, i.e. bridging cutting edge materials research with pioneering anti-mosquito PPE solutions, graphene has limited known toxicology evaluations on skin and can be costly to produce. Furthermore, graphene is not biodegradable and its black color will likely compromise the willingness to use it as a skin protector. In this work, we propose a cellulose-based approach of passive protection from mosquito bites compatible with most known active repellents. Cellulose is the most abundant polymer on earth and is the strength building block of the plant world. While it can be directly derived from the wood industry, cellulose is also immensely available in local food and paper waste, and this alone is an excellent incentive to the development of cellulose-based high performance materials. Cellulose is a biopolymer composed of both amorphous and crystalline regions. The acid hydrolysis of the amorphous region by sulfuric acid yields the formation of cellulose nanocrystals (CNCs) (cf. Fig. 1). These nanocrystals are anisotropic rods of approx. 200 nm in length and 5 nm in diameter. Their size can vary depending on their source, as plants, tunicates, and different bacteria yield different sizes (10, 11). CNCs are challengers to fossil derived materials in a wide range of industries from packaging to cosmetics. They are also highly regarded as an alternative to organics solvents in industries such as coatings and paints where the surface chemistry and size distribution allow emulsification and good dispersion. Sulfuric acid hydrolysis of the cellulose leaves negatively charged particles which limits aggregation in solution and allows CNC to be readily soluble in water, further pushing its “green” compatibility and commercial edge.

**Fig. 2.**
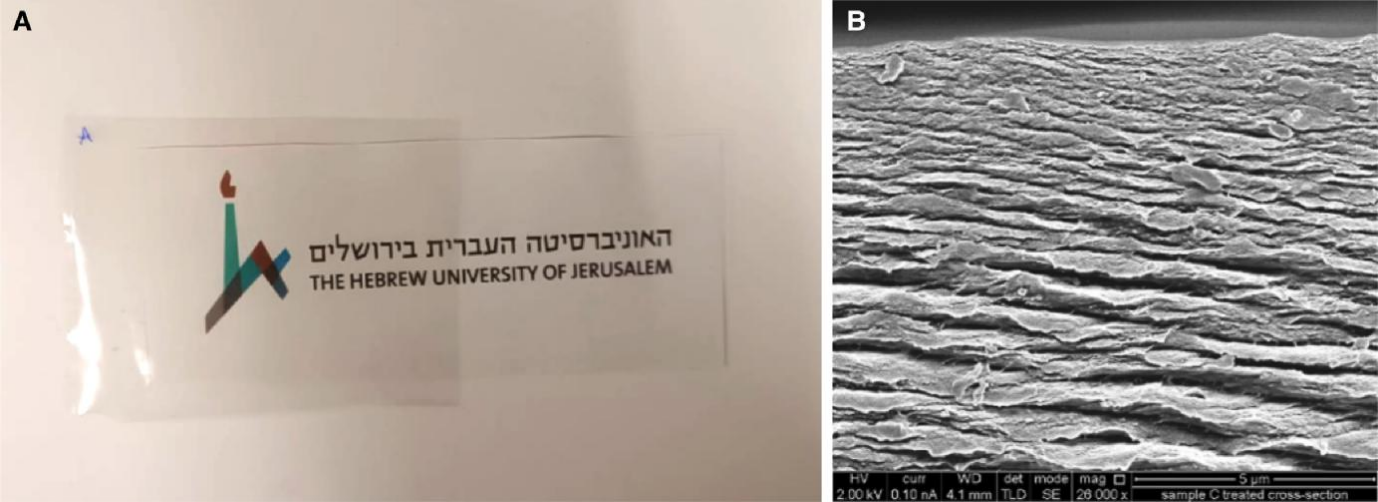
A) Self-standing transparent film of pure CNC after EISA. B) Cross-sectional view captured with extra-high resolution scanning electron microscope Magellan 400L (ThermoFisher, former field electron and ion company (FEIC)—acknowledgements are due to Dr. Ilya Torchinsky and Mr. Amir Rudich for the scanning electron microscope (SEM) images.

In summary, CNC is an affordable, sustainable material that is transparent at low concentrations and dispersed in water. It is an amphiphilic material allowing it to bind to a vast range of materials. Its surface chemistry and optical and dielectric properties make it an excellent candidate as a matrix material for high performance composites. At low concentrations in water (<10%), CNCs are transparent and can be solvent cast and dried into robust transparent thin films in a method known as evaporation-induced self-assembly (EISA) (Fig. 2) (12).

CNCs exhibit liquid-crystal (LC) behavior at these concentrations and the LC properties can be tuned with concentration, pH, drying conditions, and various additives. These features make CNC a remarkable candidate for stable dispersions and emulsions in cosmetic applications. In addition, single crystals of CNC exhibit extremely high strength, displaying a Young’s modulus comparable to that of metals (E=150 GPa) (13). CNC-based films exhibit lower but nonetheless high tensile strengths with reported E=4 GPa and reaching up to E=8 GPa with additives such as Resilin or Zein (15, 14). This work proposes to use CNC, in an analogous approach to that taken by Castilho et al., as a non-chemical camouflage shield from mosquitoes in a first stage, and assess its ability to host active repellents improving the efficiency of the overall PPE solution (16).

The biting mechanism in mosquitoes is not as simple as probing a surface and, as described by Castilho et al., cannot be simply reproduced in lab conditions (16). To overcome both the weakness of the proboscis and the different hardness of different skins, mosquitoes engage in a complex, multi-step behavior. A combination of back-and-forth motion of the maxilla and labrum together with the release of chemical compounds allows penetration in most mammalian (and even reptilian) epidermis. Mimicking penetration through a CNC membrane would be a limited model both in the selection of the probe and the probing method.

Mosquitoes also rely on multiple sensory systems, including chemical cues to detect a human host. Female mosquitoes are the only ones to feed on blood, as they source in it the nutrients required to lay eggs. They first sense a host tissue through various cues such as visual cues (contrasts), elevated temperatures, humidity, CO${}_{2}$, and other organic compounds from sweat. These cues can all be detected at once or in the absence of one or more, and back-up sensing of other cues will also be used. This further limits the ability to model and pinpoint the exact barrier mechanisms.

Mosquitoes have evolved over hundreds of millions of years. This work attempts to investigate the barrier effect of CNC on *Aedes* as they are both disease vectors. *Aedes aegypti* are daytime biters with a preference for humans over other animals. They are typical vectors of arboviruses such as dengue, chikungunya, and yellow fever. The initial results described below showed very encouraging results from the CNC formulated as a barrier on the skin. CNC’s unique surface chemistry and aspect ratio allow easy, stable dispersion of various molecules including molecules that would not disperse well in water. This prompted the investigation of the combined effect of CNC as a barrier and a safe, water-based way to disperse existing repellents. Dekel et al. recently published a work on the efficiency of indole to inhibit host attraction in *Ae. aegypti* (17). However, they have used diethyl ether as a vehicle solvent. In this work, indole is directly dispersed in the CNC–glycerol solution and the composite CNC–glycerol–indole is assessed as a barrier.

## Results and discussion

### CNC–glycerol preparations on human hand reduce *Ae. aegypti* mosquito biting

**Fig. 3.**
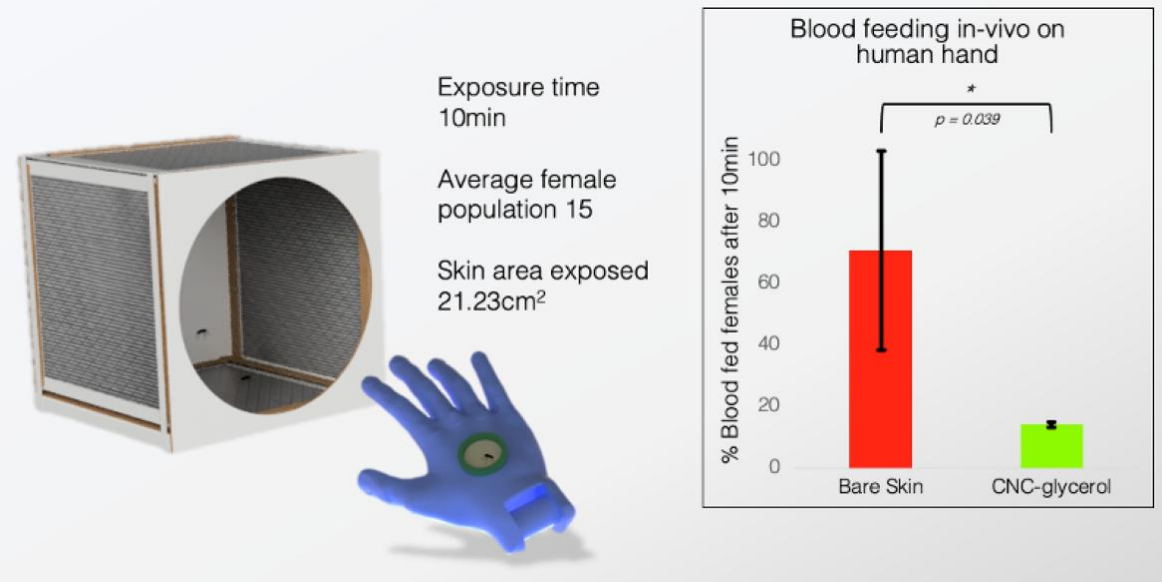
Hand testing setup and percent blood-fed females in cage with CNC–glycerol on hand and without CNC–glycerol on hand. CNC significantly reduces the blood feeding (P<0.5).

Initial testing was built on Castilho et al.’s initial approach and as the most straightforward way to evaluate the barrier effect of CNC. The author’s hand was placed in a cage with an average of 15 females for 10 min. A defined area of the skin was exposed to the mosquitoes, adapted from Dekel et al., with and without CNC on the skin as described in the experimental section (17). The first conclusion was that CNC (pH 5.5, 2 wt.% in water) did not dry ideally on the skin’s surface as its brittleness caused it to crack along the lines of the skin (cf. Fig. S2). To alleviate this issue, 0.1 wt.% of glycerol was added to the CNC aqueous solution. The CNC–glycerol was applied on the skin as a gel and let to dry for a few minutes. For both treatments (with and without CNC–glycerol), a new set of mosquitoes were used and three cages per treatment were performed. The CNC–glycerol showed a significant and dramatic reduction of blood-fed mosquitoes with 71±31% blood-fed females in nominal conditions after 10 min and only 14±1% blood-fed females when CNC–glycerol was applied on skin (cf. Fig. 3) (from this point in writing CNC when referred to as the investigated barrier refers to CNC–glycerol).

This result was encouraging enough to pursue the investigation. At this stage, the exact mechanism—whether it is chemical or physical—could not be pinpointed. On the one hand, only one human subject was tested, and the behavior of mosquitoes has been shown to be highly dependent on the individuals features as cues for mosquito sensing. In fact, Verhulst et al. show that even the skin microbiota (unique to each individual) affects the attractiveness of mosquitoes (18). On the other hand, the observation of the video monitoring of each experiment revealed that for the CNC coated hands, very few mosquitoes landed on the hand suggesting that the CNC offers a chemical barrier. Moreover, the few females that did land on CNC coated hands systematically blood-fed suggesting that the physical barrier hypothesis was not confirmed. This may be due to uneven spread of the CNC–glycerol which could be improved with cosmetic formulation. Another option is that CNC–glycerol does not fully dry on the skin and the complex probing of the mosquitoes’ proboscis allows to penetrate through by agitating the molecules and creating a path through to the skin. To further investigate the effect, a more controlled method was attempted, and an artificial feeding system was selected to feed mosquitoes over a longer period of time. Another experiment to check only the chemical barrier properties of CNC–glycerol was performed as a headspace experiment analysis the permeability of coated paper filters to ammonium hydroxide vapors as described below.

### CNC–repellent preparations on an artificial membrane reduce egg laying in *Ae. aegypti*

**Fig. 4.**
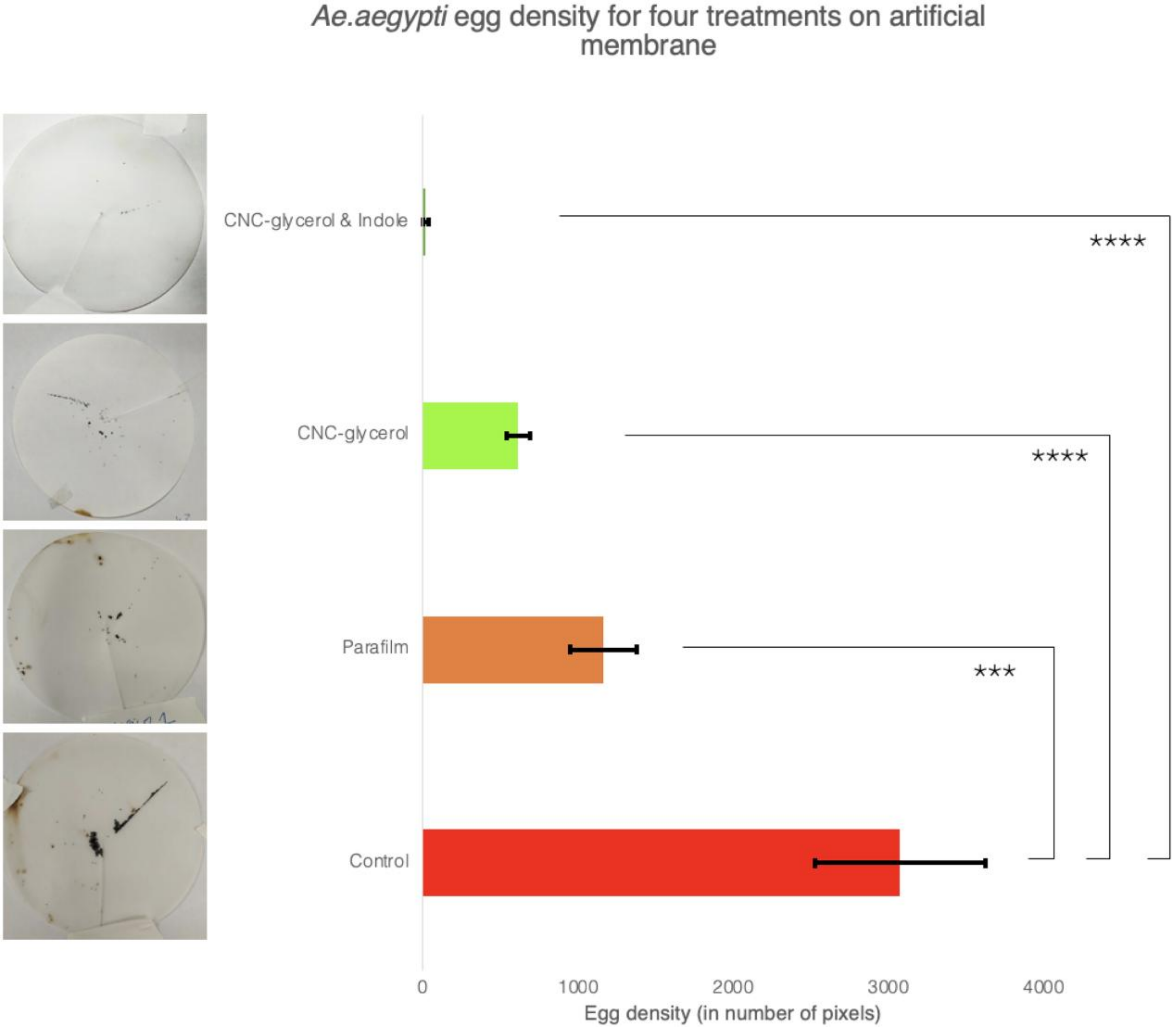
Eggs laid after Hemotek feeding on *Ae. aegypti* for 1 h through four types of membranes. A collagen membrane coated with CNC with 5 wt.% glycerol and 1mM indole, a collagen membrane with CNC–glycerol, a control collagen membrane on which Bemis Parafilm was stretched, and a control collagen membrane. The full set of collected egg images is available in Fig. S1.

The Hemotek system was used to remove the bias of the human subject and allow a control of more parameters in each cage. All three cages in each treatment were fed in parallel for an hour. The filter papers on which eggs were collected were captured with the same camera and same lighting to process the results with simple image processing tools. The sums of pixels associated to eggs on each image were averaged and compared for each treatment yielding the significant results in Fig. 4.

The reduction in eggs between the control and the CNC was dramatic and confirmed a barrier effect. The parafilm was used as a comparative dense polymer coated on the skin. An added parafilm layer did significantly reduce the number of eggs laid, as is to be expected from an added layer, making it more complex for mosquitoes to penetrate through, though not as much as CNC–glycerol. Parafilm is also petroleum derived and not produced in a way that is compatible with skin application. As mentioned earlier, this solution could also be compatible with existing active repellents. In general, these consist in active volatile compounds that inhibit host attraction. To improve further the protection provided by this CNC barrier, indole was added to the solution. In 2022, Dekel et al. report the effect of indole in inhibiting animal host attraction in *Ae. aegypti*. Indole, like most other mosquito repellents, does not disperse well in water. However, the CNCs enable the stable dispersion and the subsequent self-assembly of the CNC composite into a film may entrap the dispersed molecules between the nanometric layers of CNC and enable a sustained release.

The CNC–indole coating led to the most effective egg reduction effect, with one cage showing no egg laying at all. The Hemotek system does not reproduce all the in vivo cues. This is also why many Hemotek users prefer to rub the membrane on their skin in high sweat areas to increase the cues and make the feeding more favorable. While the human bias was mitigated when using the Hemotek system, some organic compounds can still be released from the cow blood used. In addition, the Hemotek system was kept at 37 ${}^{\circ }$C and the mosquitoes would also be able to detect the color contrasts generated from the Hemotek blood containing stub. The CNC is not a perfect barrier and other cues such as heat and color may have remained detectable through the CNC membrane. These could be dealt with mixing of other nano-particles with the CNC such as SiO${}_{2}$ to block infra-red radiation or quantum dots to camouflage the skin from the mosquitoes. The collagen membranes themselves may be a limitation to this model. They were supplied by Hemotek and are thin collagen membranes with ridges to generate rounghness for improved mosquito grip. CNC may also have a thermal and optical effect. It is known to dissipate heat fast and to self-assemble with a liquid-crystal (LC) arrangement and may even lead to birefringence and structural colors (12).

The addition of indole as an active repellent assessed and confirmed the potential synergistic effect towards a total protection solution. CNC–indole was much better at egg reduction with a dramatic 99.4% reduction in eggs. It combines both the chemical camouflage provided by the CNC and the active chemical repellent that is indole. The low molarity of the indole (1 mM) minimizes the discomfort from the indole smell and further investigation could be conducted to evaluate the minimal effective indole concentration. Dekel et al. suggest that even lower concentrations could further refrain from bad smell while maintaining repellent effect (17).

A full glycerol control treatment was not performed due to lack of applicability as glycerol will not form a film on the skin in a reasonable timeframe of use and will remain sticky on the skin. The pure indole treatment was not performed here as indole cannot be well dispersed in water. This is one of the key strength of this CNC-based formulation. In addition, the effect of solvent dispersed indole was shown by Dekel et al. (17) and was the incentive to combine the CNC with this inhibitor. Therefore, CNC appears to be an effective barrier method to mosquito egg laying. Egg laying is a result of bloodfeeding and so it can be an indicator of biting and feeding effectiveness. Thus, combining the observations in the human hand experiment and the Hemotek experiment suggests that CNC could be an effective barrier to mosquito biting. While not being a perfect barrier, the combination of CNC with active repellents would be an excellent route to pursue. The intrinsic properties of the CNC allow efficient dispersion of the indole and could be an ideal method for aqueous-based dispersion and slow release mechanism for active repellents that in general require organic solvents to be effective. The efficient dispersion of indole was assessed by the lack of phase separation or pellet formation after high-speed centrifugation (10,000 rcf). These results seem to point to CNC’s potential in reducing mosquito bites and act as a platform to disperse active repellents in water.

While these results are promising, they are not sufficient to pinpoint the exact mechanism of protection. Similarly, Castilho et al. motivated by the hypothesis of graphene as a physical shield were not able to pinpoint the exact mechanism and proposed a mechanical model to assess a self-standing graphene film (16). In this work, the initial in vivo data suggested that fewer mosquitoes are attracted to land on the skin and therefore the chemical barrier hypothesis is favored. Behavioral studies of mosquitoes have identified key volatile organic compounds (VOCs) perspiring through human skin which attract mosquitoes. In particular, Smallegange et al. have proposed using a base mixture of ammonia and lactic acid as an attractant in multiple publications (20, 19). Gas barrier of CNC composite films has previously been shared (22, 21, 23). Thus a simple headspace experiment was designed to assess the ammonia barrier properties of a CNC coating. A Whatman filter paper membrane was used as an analog permeable membrane. The VOC sensor used could not detect lactic acid, so the model was simplified to ammonium hydroxide in using the rates and concentrations adapted from Smallegange to match the flow of ammonia exiting skin (19). The experiment confirmed that CNC blocks the permeation of ammonium hydroxide and further points to a chemical barrier effect. This experiment is only a model and ammonium hydroxide is one of many attractants. This could be further explored by performing in vivo with different attractants mixed in a wind tunnel for example. It is also a timed experiment where the vapors were let to permeate through the membrane for an hour in a fully static way. Both mosquitoes and humans are dynamic and mobile systems but a barrier to ammonium hydroxide was observed and quantified in the concentration, time, airflow, and volumes specified in the methods.

## Materials and methods

### Aedes aegypti

The *Ae. aegypti* were grown as per Bohbot’s et al. methodology subjected to 12 h daylight and 12 h dark environment cycles while being fed with 10 w/w% sucrose solution in water through a cotton ball (17). Dried *Ae. aegypti* eggs were hatched in water and fed with crab food through their larval phase in the same environmental conditions as the mature mosquitoes. Pupae were collected and placed in developmental cages to reach maturity with sucrose feed changed every 48 h. Mature mosquitoes were transferred to experimental cages and split according to the needs of each treatment. Table 1 outlines the population in each cage for the artificial feeding experiments of *Ae. aegypti*.

**Table 1.** Cage population for Hemotek testing on *Ae. aegypti*.

Cage reference	Females	Males
Control 1	16	18
Control 2	8	8
Control 3	15	5
CNC 1	11	9
CNC 2	14	7
CNC 3	14	18
CNC + 1 mM indole 1	12	17
CNC + 1 mM indole 2	24	4
CNC + 1 mM indole 3	17	5
Parafilm 1	11	5
Parafilm 2	14	9
Parafilm 3	10	7

For the in vivo experiments on human hand, the mosquitoes were frozen immediately after the experiment allowing monitoring of blood swelling in females thus improving the count. For the artificial membrane (Hemotek) experiments, the mosquitoes were fed *ad-libitum* for 1 h in the lab environment (20–23 ${}^{\circ }$C and a relative humidity between 40% and 50%) and immediately placed back in the incubators with a 10 wt.% sucrose solution replaced every 24 h to feed on.

## CNC preparation

In a preliminary stage, pure CNC (2 wt.%) in water supplied by Melodea (Israel) was used, titrated with sodium hydroxide (NaOH) up to pH 5.5, the average skin pH. However, this formulation cracked on the skin upon drying along the cracks of the skin (cf. supplementary information). The problem was mitigated by adding 0.1 vol.% glycerol as a plasticizer to the solution to reach 5 wt.% of the dry formulation.

### CNC–indole preparation

To assess the synergistic effect of CNC as a passive barrier together with an active repellent, indole was dispersed in the CNC glycerol solution. One millimolar of indole was added as a dry powder in the previously described solution. Ultrasonication was used to disperse the indole in a QSONICA sonicator (500 W; 20 kHz, 1 s on; 1 s off frequency) for 5 min at 50% amplitude. Indole was purchased from Sigma-Aldrich. Air bubbles were removed by centrifugation for 3 min at 5,000 rcf.

### Human hand testing apparatus

A 3D-printed ring made of two interlocking parts was clipped on a disposable nitril glove, below and above the glove, and the inner section of the glove was cut out to reveal a constant and consistent testing area. Mosquitoes were starved 1 h prior to experimenting. Experiments were conducted between 09:00 and 12:00 as this is a known window of high activity for the *Ae. aegypti*. at a temperature between 20 ${}^{\circ }$C and 23 ${}^{\circ }$C and a relative humidity between 40% and 50%. Lighting and shading were kept constant. Subject kept left hand immobile in the cage for 10 min in each treatment. Said subject had no adverse reaction to the biting, hence swollen spots were not a valid data point. A camera placed above a transparent face of the cage was used to monitor number of visits. The total exposed skin area was 21.23 cm${}^{2}$ not accounting for the curvature of the hand below. Mosquitoes were frozen to allow counting of blood fed as observed by red body swelling in females. Data analysis was done by monitoring the videos for each sampling and counting the number of visits as well as counting the visibly blood-fed insects by freezing the cages immediately after 10 min of exposure. The protocol was approved by the Committee for Research Involving Human Subjects, The Robert H. Smith Faculty for Agriculture, Food and Environment (Approval 17.2022).

### Artificial membrane feeding (Hemotek)

Unlike the human hand experiment, the artificial membrane was left for 1 h feeding in slightly larger cages. This may have allowed the insects more time to assess less obvious cues such as the heat from the Hemotek. The Hemotek was filled with 1.5 ml of cow blood obtained from the local slaughterhouse. The collagen membranes supplied by Hemotek were always used, as indicated by the supplier. When CNC and CNC–indole were applied on the membranes, the membranes were already placed on the feeders and left to dry in open air. Parafilm (Bemis) controls were prepared by stretching parafilm on top of the collagen membrane to obtain a comparative for the behavior of a dense polymer on top of the collagen membrane. For the *Ae. aegypti*, 2% adenosine triphosphate (ATP) was added to the blood as a phagostimulant. Egg collection was done by placing a Whatman filter paper on the surface of a water cup that the eggs laid on the surface could be collected simply by lifting the filter paper. The timing of the oviposition cup varied with the species. For the *Ae. aegypti*, egg-laying cups were placed 48 h after blood feeding. The difference in mass between cages was too subtle to accurately measure whereas the dark color of the eggs allowed easy optical counting. The number of eggs was too high for manual counting and the contrast between the eggs and other features in the image enabled to identify the grayscale threshold corresponding to eggs in the image. The threshold was determined using the image processing toolbox in Matlab and the number of pixels on the grayscale below the determined threshold was summed using FIJI image analysis software and the histogram tool. This method was used as it offered a high throughput. Weighing the eggs was discarded as the mass difference was too low to determine with sufficient accuracy and the transfer of the membranes to the scale would cause loss of material. The statistically significant results are a promising direction. In future work, the video monitoring of the membranes combined with image processing tools could enable further parameters to be taken into consideration such as the frequency of visits and the average landing time. These methods were not available for this experiment.

### Ammonium hydroxide barrier headspace experiment

**Fig. 5.**
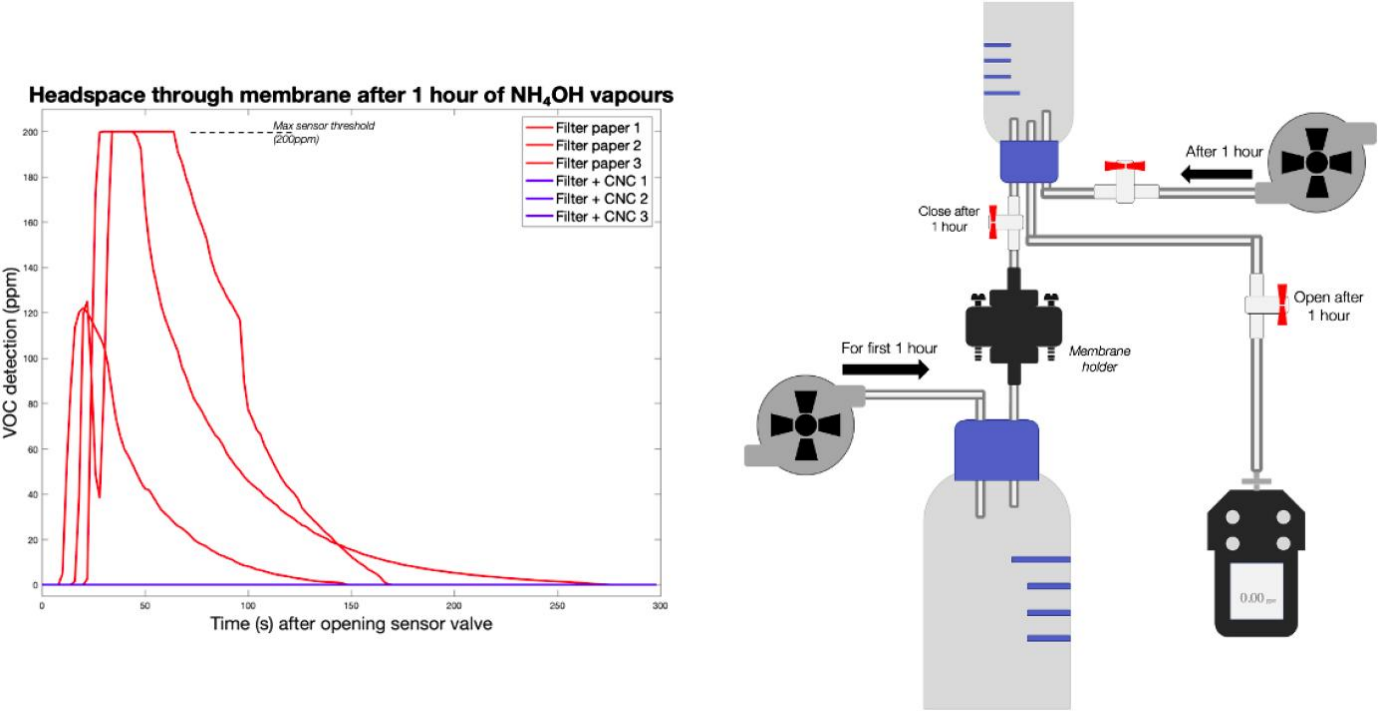
Headspace VOC analysis of 250 ml after 1 h of air flow in ammonium hydroxide (10 ml of 2.5%) through a filter membrane. Left is the VOC reading for three repeats of a plain membrane (all detections above 100 ppm) and three repeats through a CNC–glycerol coated membrane (all readings at 0 ppm throughout the time of reading). Right is a schematic representation of the headspace setup.

To assess the chemical barrier hypothesis, a simple headspace experiment was designed by adapting volatile usage and flow from Smallegange et al. (19). The designed setup is illustrated in Fig. 5. In this experiment, a 2.5% ammonium hydroxide was prepared by diluting 1 ml of 25% ammonium hydroxide (Arcos Organics) in 9 ml of distilled water and placed in a 500 ml glass bottle. The cap was perforated to fit two tubes sealed with hot glue and parafilm. Leakage was assessed for each connecting element with two flow-meters. One fitting was used as an inlet for a pump and the second was connected to a membrane holder. The membrane holder was designed using Solidworks computer aided design (CAD) modeling software (Dassault Systems) and 3D printed on an Ender-3 printer (Creality) using poly-lactic acid (PLA) filament (Spider 3D, Israel). The printed parts were sealed with parafilm (Bemis). Both sides of the membrane holder were identical and a silicon O-ring was fitted above the membrane to isolate it. The membrane holder was connected to a second glass bottle (250 ml) through a tap. Two other taps were fitted on the second container. One was used for in-flow of air and the second was an outlet directed to a WatchGas Poli MP400 VOC sensor (WatchGas detection). The taps allowed to circulate vapors for the first container through the membrane into the second sealed container for 1 h. After 1 h, the tap above the membrane was closed and the two other taps open allowing the headspace content to flow through to the VOC sensor. A control treatment was repeated (n=3) with a Whatman 1 filter membrane (Whatman). To assess the CNC–glycerol barrier properties, fresh solutions of CNC–glycerol were prepared as described above (5 dry wt.% of glycerol was added to a 2 wt.% solution of CNC in water, sonicated and centrifuged). Whatman 1 filters (47 mm diameter) were coated with 2 ml of the CNC–glycerol solution and let to dry. The experiment was repeated (n=3), and control and treated membranes were run in a random order. A fresh solution of ammonium hydroxide was prepared for every repeat. The airflow used in both steps of the experiment was maintained at 500 ml/min. After 1 h, the VOC sensor data were collected using the WatchGas Suite software. One point was read every 2 s for 5 min until all readings were 0.00 ppm. In some cases, the measured VOC content exceeded the max threshold of the sensor (>200 ppm). In all three CNC treatments, the reading was consistent at 0.00 ppm. The content may have been lower than the resolution of the sensor and this is to be taken into consideration in the result analysis.

## Conclusion

CNC was found to reduce the blood feeding in *Ae. aegypti* when tested on a single human hand and on an artificial feeding system by assessing the eggs laid after feeding *Ae. Aegytpi* with and without CNC and confirmed that CNC can act as a chemical camouflage significantly reducing the laid eggs counted, and thus the number of blood-fed females per cage. The combined camouflage effect of CNC and the active repelling of indole further reduced egg laying and confirmed the excellent potential of CNC as a media for active repellents produced in a safe and sustainable way. CNC’s chemical barrier effect was shown in a headspace experiment where ammonium hydroxide, a known mosquito attractant, was blocked by the CNC–glycerol coating. The biocompatibility of the CNCs, their ubiquity, as well as the self-assembly characteristic together with the ability for cost effective mass production makes CNC ideal for the development of a new generation of mosquito PPE.

## Supplementary Material

pgad069_Supplementary_Data

## Data Availability

All data are included in the manuscript and/or supporting information.

## Previous Presentation

No previous presentation.
