# Supplementary material for: Mosquito bite prevention through self-assembled cellulose nanocrystals
Source: PNAS Nexus. 2023 Apr 11;2(4):pgad069. doi: 10.1093/pnasnexus/pgad069 (PMC10089072; doi:10.1093/pnasnexus/pgad069)
Supplement: pgad069_Supplementary_Data [file pgad069_supplementary_data.zip › PNASNEXUS-PNASNEXUS-2023-00165-T-s01.docx]

Supplementary Information to: Mosquito Bite Prevention through Self-Assembled Cellulose Nano Crystals

Daniel Voignac, Evyatar Sar-Shalom, Yossi Paltiel, Oded Shoseyov, and Jonathan Bohbot


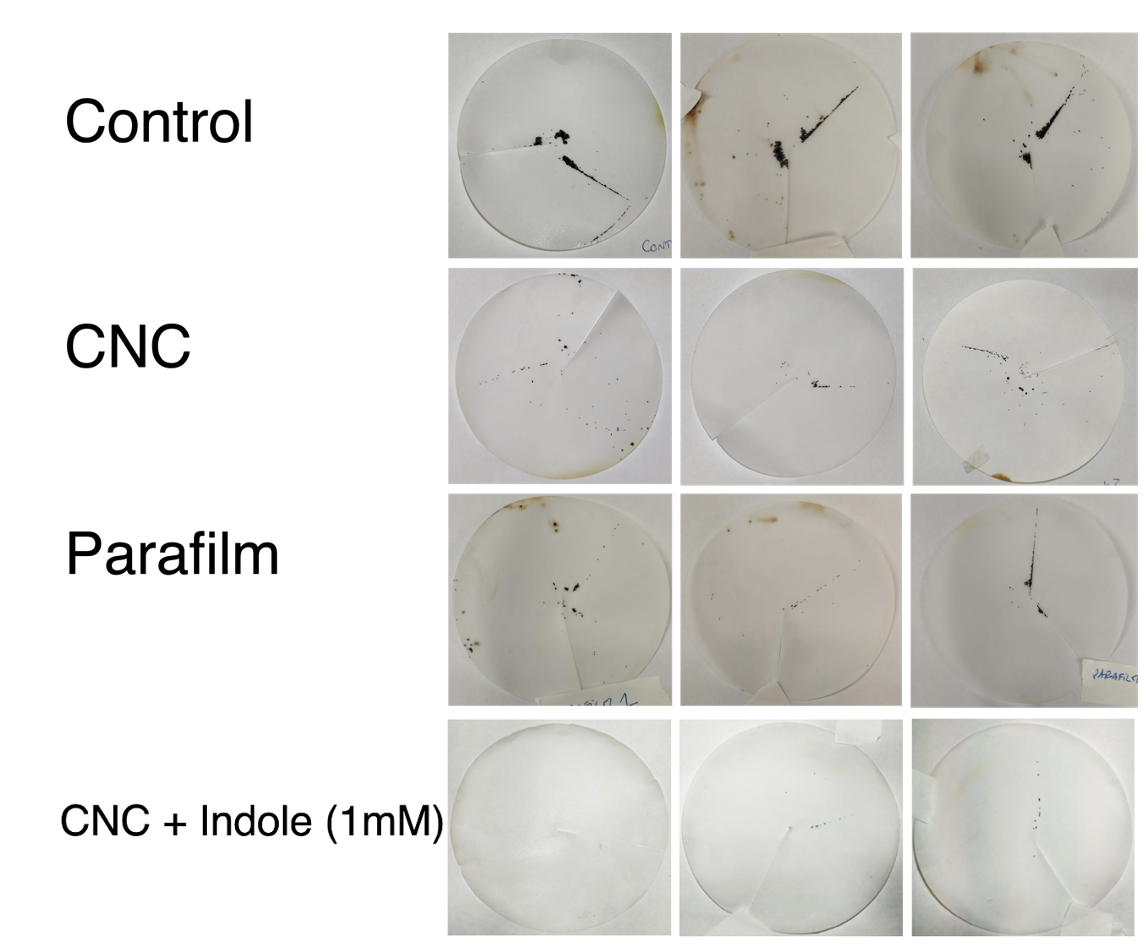


Figure S1 Eggs laid after Hemotek feeding on Ae.Aegypti for 1h through four types of membranes. A collagen membrane (top row), a collagen membrane coated with CNC with 5wt% glycerol (second row), a stretched Bemis Parafilm (third row), CNC glycerol and 1mM Indole (bottom row)


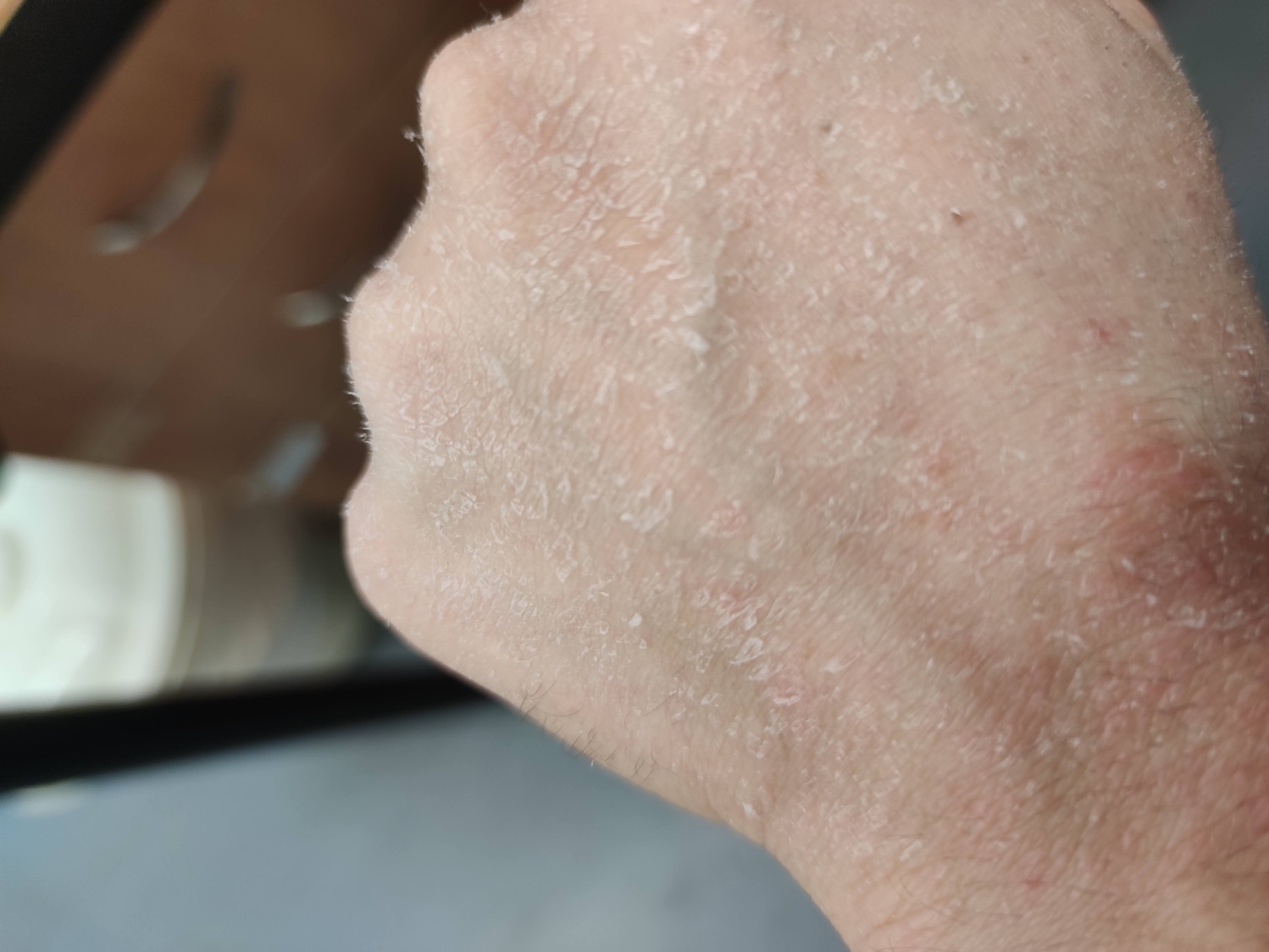


Figure S2 Close-up image of the pure CNC (pH 5.5) on human skin showing a cracked dry film with cracks following the skin’s pattern
